# Circadian lipid and hepatic protein rhythms shift with a phase response curve different than melatonin

**DOI:** 10.1038/s41467-022-28308-6

**Published:** 2022-02-03

**Authors:** Brianne A. Kent, Shadab A. Rahman, Melissa A. St. Hilaire, Leilah K. Grant, Melanie Rüger, Charles A. Czeisler, Steven W. Lockley

**Affiliations:** 1 Division of Sleep and Circadian Disorders, Departments of Medicine and Neurology, Brigham and Women’s Hospital, Boston, MA USA; 2 Division of Sleep Medicine, Harvard Medical School, Boston, MA USA; 3 Department of Psychology, Simon Fraser University, Burnaby, BC Canada

**Keywords:** Circadian mechanisms, Circadian regulation

## Abstract

While studies suggest that light and feeding patterns can reset circadian rhythms in various metabolites, whether these shifts follow a predictable pattern is unknown. We describe the first phase response curves (PRC) for lipids and hepatic proteins in response to combined light and food stimuli. The timing of plasma rhythms was assessed by constant routine before and after exposure to a combined 6.5-hour blue light exposure and standard meal schedule, which was systematically varied by ~20° between individuals. We find that the rhythms shift according to a PRC, with generally greater shifts for lipids and liver proteins than for melatonin. PRC timing varies relative to the stimulus, with albumin and triglyceride PRCs peaking at a time similar to melatonin whereas the cholesterol and high-density lipoprotein PRCs are offset by ~12 h. These data have important implications for treating circadian misalignment in shiftworkers who consume meals and are exposed to light around the clock.

## Abstract (web summary)

A key property of circadian rhythms is that they can be reset in response to environmental time cues; this response is described by a Phase Response Curve (PRC). Here the authors describe PRCs for resetting circadian rhythms in lipids and hepatic proteins in response to combined light and food exposure.

## Introduction

While studies suggest that light and feeding patterns can reset circadian rhythms in various metabolites, whether these shifts follow a predictable pattern is unknown. Here, we describe the first phase response curves (PRC) for lipids and hepatic proteins in response to combined light and food stimuli. The timing of plasma rhythms was assessed by constant routine (CR) before and after exposure to a combined 6.5-h blue light exposure and standard meal schedule, which was systematically varied by ~20° between individuals. The rhythms shifted according to a PRC, with generally greater shifts for lipids and liver proteins than for melatonin. PRC timing varied relative to the stimulus, with albumin and triglyceride PRCs peaking at a time similar to melatonin whereas the cholesterol and high-density lipoprotein PRCs were offset by ~12 h. These data have important implications for treating circadian misalignment in shiftworkers who consume meals and are exposed to light around the clock.

Circadian rhythms in physiology, metabolism and behaviour are driven by a multioscillatory system, in which the suprachiasmatic nucleus (SCN) is the retinorecipient central circadian pacemaker critical for synchronising circadian rhythms to daily light–dark cycles. Under controlled conditions, the daily rhythms of core-body temperature, and of melatonin and cortisol secretion are considered robust markers of the timing of the central circadian pacemaker^1^. In addition, there are circadian clocks located in peripheral organs and tissues that are thought to refine the timing of local physiological systems in concert with the central pacemaker and are reset by both photic and non-photic time cues in animal models^2^. To date, there is limited evidence for endogenous peripheral clocks in humans. While significant day-night^3–6^ and endogenous circadian rhythms^6–11^ in a range of metabolites have been documented, it is unknown whether these metabolic rhythms are controlled by peripheral clocks, the central pacemaker, or both.

Light is considered the most powerful time-cue (zeitgeber) for resetting the central circadian pacemaker in humans, as assessed using markers such as melatonin, cortisol, and core-body temperature^12^. Non-photic time cues such as exercise or mealtimes can also reset circadian rhythms of these markers in humans, but are much weaker stimuli than light^10,13–17^. Interestingly, in rodents, peripheral clocks can shift independently from the light schedule, uncoupling from the SCN and aligning with mealtime under time-restricted feeding schedules^18,19^. Whether such uncoupling occurs in humans is not well characterised^20^. Recent evidence suggests that timed meals play a role in synchronising plasma glucose rhythms and core clock gene expression in adipose tissue^15^ and that metabolite rhythms can be shifted out of alignment with central circadian markers by behavioural time cues (e.g., sleep/wake and feeding/fasting cycles)^10^.

The direction and magnitude of circadian phase resetting in response to a stimulus are described by a PRC. The degree of phase shift in response to light is affected by the timing, intensity, duration, and wavelength of the light stimulus. Previously, we described the PRC for shifts in the centrally controlled melatonin rhythm to either 6.7 h or 1 h of white light^21–23^, and the respective dim light controls^23^, thereby isolating the effects of light from meal timing, which was identical between conditions. More recently, we constructed a PRC to a 6.5 h blue (480 nm) light stimulus [the peak sensitivity of the melanopsin-containing intrinsically photosensitive retinal ganglion cells (ipRGCs) that primarily mediate photic circadian resetting^24^], using the same protocol^25^, and found maximal phase resetting to be similar, at ~3 h.

In the present analysis of the same blue light study, we investigated the phase-resetting effects of the stimulus on peripheral rhythms in circulating lipids and clinical markers of hepatic function. As in the white light PRC studies, the stimulus was a combination of the 6.5-h light pulse and a standard meal schedule (three meals and a snack). Sixteen healthy participants (18–30 years; 8F) were studied for 9–10 days in an environment free of time cues. Endogenous circadian rhythms in these markers were assessed under CR conditions, which included constant wake in dim light, continuous semi-recumbent posture, and identical isocaloric snacks and fluids every hour. This protocol removes or evenly distributes environmental stimuli, such as sleep-wake, rest-activity, and feeding-fasting cycles, that may potentially mask the endogenous circadian rhythm^26^.

**Fig. 1:**
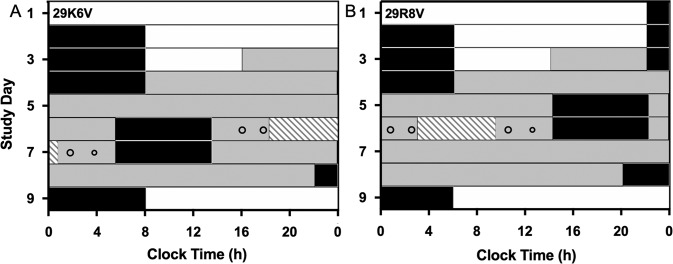
Study protocol. The schedule for participants (**A**) 29K6V and (**B**) 29R8V, plotted in raster format. White and grey bars indicate wake episodes in ambient light of <190 lux or <3 lux, respectively. Black bars indicate scheduled sleep in darkness (0 lux). The hatched bars indicate the 6.5-h light exposure (LE). The open circles indicate the timing of meals and snacks (smaller circle) during the exposure day.

Following three inpatient baseline days (8:16 h sleep:wake), participants underwent a 30- to 52-h CR (CR1) to assess initial circadian phase, the combined light exposure (LE)/mealtime day preceded and followed by an 8-h sleep episode, and then a second 32- to 55-h CR (CR2) to assess any resultant shifts in circadian rhythms (Fig. 1). The 16-h combined light/meals exposure day was identical between individuals except that the timing was systematically varied to schedule the midpoint of the day 80 min (~20°) apart between participants to collectively cover all circadian phases. Breakfast, lunch, dinner, and a snack were served at 2.67, 4.42, 12.42, and 14.42 h after waking, respectively—an 11.75-h feeding window—and the 6.5-h 480 nm LE was scheduled from 4.75 to 11.25 h after waking.

## Results

### Circadian rhythm assessment

**Fig. 2:**
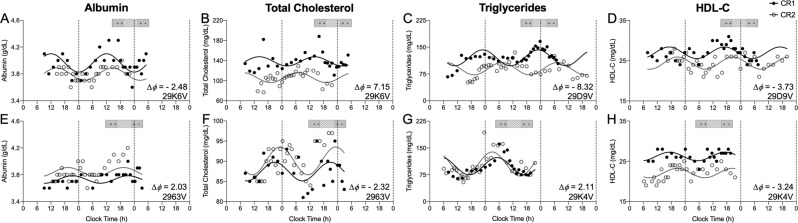
Examples of rhythms in CR1 and CR2. Data collected during CR1 (black filled circles) and CR2 (grey open circles) for (**A**, **E**) albumin, (**B**, **F**) total cholesterol, (**C**, **G**) triglycerides, and (**D**, **H**) HDL-C. The *x*-axis is the time each blood sample was taken and the *y*-axis is the clinical assay concentration. The solid black line is the fitted cosinor regression for CR1 and the grey solid line is the fitted cosinor regression for CR2. The phase shift (Δϕ) and participant ID for each example is noted in the bottom right corner of the plots. The exposure day schedule is represented in the bar above the plots; grey bars indicate wake episodes in ambient light <3 lux, hatched bars indicate the 6.5-h light exposure (LE), and the open circles indicate the timing of meals and snacks.

First, data from CR1 and CR2 were fit separately with 24-h sinusoidal functions to establish endogenous circadian rhythmicity and determine the peak time (acrophase) of the rhythms (Fig. 2 and Supplementary Fig. 1)^27^. Phase shifts were calculated for each participant for each parameter individually as the change in acrophase clock time between CR1 and CR2. Only significant sinusoidal fits (amplitude different from zero, two-tailed, *p* < 0.1) were used to estimate acrophase and calculate phase shifts. During CR1, among the 16 participants studied, significant 24-h rhythms were detected in total protein (*n* = 11), albumin (*n* = 7), globulin (*n* = 9), total cholesterol (*n* = 9), triglycerides (*n* = 13), low-density lipoprotein cholesterol (LDL-C) (*n* = 9), and high-density lipoprotein cholesterol (HDL-C) (*n* = 9) (Table 1 and Supplementary Table 1, Supplementary Fig. 2). During CR2, significant 24-h rhythms were detected during CR2 in total protein (*n* = 14), albumin (*n* = 12), globulin (*n* = 11), total cholesterol (*n* = 8), triglycerides (*n* = 16), LDL-C (*n* = 13), and HDL-C (*n* = 14).

**Table 1:** Summary of endogenous rhythmicity and phase shifts.

Class	Assay	24 h rhythms under CR1 (count, %)	24 h rhythms under both CR1 and CR2 (count, %)	Mean acrophase CR1 (decimal time ± SD)	Max delay (h)	Max advance (h)
	Melatonin	16/16 (100)	16/16 (100)	3.41 (±1.41)	−2.59	1.52
Hepatic proteins	Total Protein	11/16 (69)	10/16 (63)	16.32 (±3.95)	−5.46	7.10
	Albumin	7/16 (44)	6/16 (38)	15.81 (±5.01)	−5.05	7.12
	Globulin	9/16 (56)	6/16 (38)	17.64 (±3.60)	−1.49	7.99
	Total Cholesterol	9/16 (56)	7/16 (44)	18.32 (±2.35)	−2.32	7.15
Lipids	Triglycerides	13/16 (81)	13/16 (81)	3.22 (±2.92)	−8.32	2.11
	LDL-C	9/16 (56)	8/16 (50)	16.59 (±2.20)	−3.63	6.76
	HDL-C	9/16 (56)	8/16 (50)	19.17 (±1.75)	−3.73	4.60

### Constructing phase response curves

**Fig. 3:**
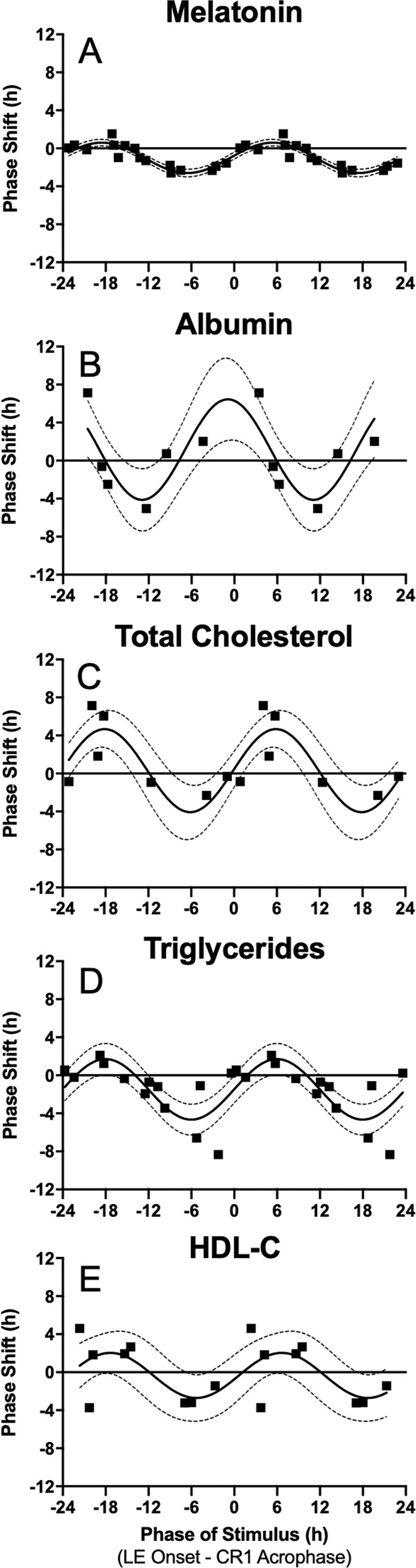
Phase response curves (PRCs). Raw phase shifts in (**A**) melatonin, (**B**) albumin, (**C**) total cholesterol, (**D**) triglycerides, and (**E**) HDL-C plotted as a function of circadian phase of the stimulus, defined as the onset of LE - CR1 assay-specific acrophase. PRCs are double-plotted. The solid horizontal black line indicates no phase shift. Black squares represent each individual that had statistically significant cosinor regressions in the assay during both CR1 and CR2. The solid sinusoidal black line is the fitted single-harmonic function. The 95% confidence intervals generated from the fit single-harmonic function are shown by the grey dotted lines.

Next, to construct the PRCs, the timing of the stimulus (defined as the timing of LE onset during the exposure day) was expressed relative to the acrophase of each parameter during CR1 (abscissa) and plotted against the phase shift observed (ordinate) for phase advances (shifting earlier, positive value) or phase delays (shifting later, negative value) per convention (Fig. 3). There were statistically significant PRCs for melatonin (inter-individual range of shifts −2.59 to 1.52-h, nonlinear regression overall fit *p* = 0.0001), albumin (−5.05 to 7.12-h, *p* = 0.016), total cholesterol (−2.32 to 7.15-h, *p* = 0.0013), triglycerides (−8.32 to 2.11-h, *p* = 0.0004), and HDL-C (−3.73 to 4.60-h, *p* = 0.042). Shifts in total protein, globulin, and LDL-C did not exhibit a PRC (*p* > 0.05; Supplementary Fig. 3).

To assess whether the variance in the resetting response was better explained when modelled relative to the phase of the central pacemaker (rather than relative to the phase of each respective parameter separately), PRCs were also constructed defining circadian phase of the stimulus relative to the melatonin rhythm, assessed by the timing of dim light melatonin onset (DLMO), a reliable marker of the central pacemaker. Constructing PRCs relative to DLMO also allowed direct comparison to the PRC for shifts in melatonin^25^. This approach did not improve the PRC fits in general: The r^2^ was higher when PRCs were constructed using the initial circadian phase of the stimulus to determine exposure timing for melatonin (0.80 vs. 0.76), albumin (0.60 vs. 0.07), total cholesterol (0.70 vs. 0.25), and triglycerides (0.50 vs. 0.50), but not HDL-C (0.39 vs. 0.61) (Supplementary Table 2). To assess coupling of the central and putative peripheral rhythms, phase shifts of melatonin were compared to the phase shifts in the lipid and hepatic markers. There were no statistically significant correlations between phase shifts of melatonin and the phase shifts of the other parameters (p > 0.05) (Supplementary Fig. 4). There were also no significant changes in parameter amplitude between the two CRs when using z-scored data for albumin, total cholesterol, or HDL-C, but there was a lower amplitude during CR2 for triglycerides (paired t-test, *p* = 0.02) (Supplementary Fig. 5). The degree of phase shift was not associated with changes in amplitude for any of the markers (*p* > 0.05; Supplementary Fig. 5).

### Phase response curves adjusted to relative clock time

Finally, to enable a direct comparison of PRC timings, the PRCs were re-plotted on the same scale, using the clock time of the group average CR1 acrophase as ‘0’ for the timing of the stimulus (i.e., 0 representing when light onset is coincident with the acrophase for the group during CR1, and realigned along the x-axis to the corresponding clock time) (Supplementary Fig. 6).

## Discussion

While the robust entraining effects of light on the central circadian pacemaker have been clearly established, little is known about the effects of photic and non-photic stimuli on the regulation of circadian rhythms of peripheral metabolic markers in humans. In this study, we evaluated the effects of a combined photic and non-photic stimulus on clinical plasma markers of hepatic function and lipids, specifically total protein, albumin, globulin, total cholesterol, triglycerides, LDL-C, and HDL-C. Similar to melatonin, phase resetting of albumin, total cholesterol, triglycerides, and HDL-C followed a type 1 PRC^25^, but the magnitude and direction of shifts differed from melatonin, suggesting different control and phase-resetting properties than markers of the central circadian pacemaker.

The metabolic rhythms were less consistently rhythmic than melatonin, although a majority of individuals exhibited significant rhythms in total protein, globulin, total cholesterol, triglycerides, LDL-C, and HDL-C when analysed using the typical cosinor model. The CR protocol is the gold standard technique for defining internally generated circadian rhythms and their presence provides definitive evidence for endogenous rhythms in these clinical markers. This finding has important clinical implications as this natural circadian variation is not taken into account when assessing laboratory test results, which may confound clinical decisions that are often based on normative data derived from a single time point^28^.

The current protocol cannot conclusively determine whether these rhythms are under the control of the central and/or peripheral clock(s), or whether light or meal timing is the principal resetting signal. The observation that liver function and lipid rhythms do not shift in parallel with the centrally controlled melatonin rhythm, and exhibit larger phase shifts, suggests that they may be separately regulated. Other centrally controlled markers, such as core-body temperature and cortisol, have the same endogenous period and a stable phase relationship with melatonin^29,30^, and shift in parallel with melatonin in response to light^21,31,32^. These findings suggest that these peripheral rhythms are not just more ‘hands’ of the central clock but represent separately generated and regulated peripheral rhythms, as confirmed in in vivo and in vitro studies in animal models, including in the liver^19,33,34^. We postulate that the same is true in humans.

While in rodents there is compelling evidence that peripheral tissues can generate endogenous rhythmicity independent from the SCN in vitro and in vivo^35,36^, in humans there is limited evidence for peripheral clock rhythms functioning independently and being shifted in a different direction, and to a different degree, than central clock markers (e.g., melatonin). The current data suggest such a functional separation, which we hypothesise is driven independent of the SCN by peripheral clocks. There is some evidence from animal studies that PRCs differ in response to photic and non-photic stimuli^37^ and that peripheral clocks can be reset according to a PRC^38^. Conversely, it may also be possible that differences in PRCs could result from the interaction between the SCN clock and other damped oscillators, or even uncoupling within the SCN clock itself.

Notwithstanding the source of the rhythmicity, the construction of type 1 PRCs from changes in phase between two CRs is clear evidence that these rhythms are under circadian control, and that they meet two canonical properties of a circadian rhythm: (i) are rhythmic in the absence of external time cues (demonstrated by CR1); and (ii) can be reset in response to a stimulus in a predictable manner (demonstrated by the PRCs). Furthermore, the magnitude of the Type 1 phase shifts in liver function and lipid profile markers are much greater, up to 8 h, compared to the ~3 h typically observed for central markers in response to light^22,25,39^.

Understanding the relative timing of the PRCs is complex given the large differences in the acrophase times between the parameters and the multifaceted stimulus (light and meal timing). Comparing the approximate clock time of the ‘0’ circadian phase exposure (where light onset is coincident with the parameter acrophase for the group during CR1), shows that the melatonin and triglyceride PRCs have similar timing (delay shifts in the ~12 h before their early morning acrophase) whereas total cholesterol and HDL-C profiles different by ~12 h (delay shifts in the 12 h before their late afternoon acrophase). Future studies that isolate the effects of either lighting or mealtimes are needed to determine which time cues each parameter is most sensitive.

To summarise, plasma markers of hepatic function and lipids show endogenous circadian rhythms that shift in response to a combined light and meal schedule. The direction and magnitude of the shifts in albumin, total cholesterol, triglycerides, and HDL-C can be described by conventional type 1 PRCs that are distinct from the PRC for melatonin. Further work is needed to demonstrate whether food or light is the main environmental time-cue resetting each peripheral marker. Understanding the temporal dynamics of peripheral circadian rhythms is critically important for designing behavioural and clinical approaches using photic and non-photic interventions to treat the circadian misalignment associated with shiftwork, jetlag, and disease^40^.

## Methods

### Ethical approval

The study was approved by the Brigham and Women’s Hospital through the Partners Human Research Committee, in compliance with the Declaration of Helsinki. All participants gave written informed consent prior to enroling in the study and were paid for their participation.

### Participants

Twenty-one participants (10F, mean age ± SD: 23.10 ± 3.43 years) were studied for 9–10 days in the Intensive Monitoring Unit (IPM) of the Center for Clinical Investigation (CCI) at Brigham and Women’s Hospital, Boston, MA between May and December 2009.

### Experimental protocol

During the 9–10 day protocol, participants remained in an individual time-free suite. The protocol started with three baseline days consisting of 8 h of scheduled sleep in darkness (<0.02 lux, <0.00006 W/m^2^) and 16 h of scheduled wake in ambient light. Ambient light was provided by 4100 K fluorescent lamps (Philips Lighting, The Netherlands) with digital ballasts (Lutron Electronics Co., Inc, PA) transmitted through a UV-stable filter (Lexan 9030 with prismatic lens, GE Plastics, MA) and light levels were ~90 lux [0.23 W/m^2^ (~89 lux) at 137 cm in the vertical plane and 0.48 W/m^2^ (~190 lux) in the horizontal plane at a height of 187 cm]. Halfway through Day 3, the light intensity was dimmed to ~0.5 lux (0.001 W/m^2^) at 137 cm from the floor in the vertical plane with a maximum <3 lux (0.01 W/m^2^) at 187 cm from the floor in the horizontal plane anywhere in the room for the remainder of the study. Participants were in darkness during scheduled sleep. At wake time on Day 4, participants began an initial ~30–52-h CR in <3 lux during which time participants remained awake in a semi-recumbent position in bed and were provided equal isocaloric snacks at hourly intervals while being constantly monitored by a technician to maintain wakefulness. Following an 8-h sleep episode, participants were exposed to the photic stimulus, which was monochromatic 480-nm light (11.8 μW/cm^2^; 2.8 × 10^13^ photons/cm^2^/s; ≤15-nm half peak bandwidth) for 6.5 h via a modified Ganzfeld dome^25^ centred in the 16-h wake episode, and the non-photic stimulus, which included meals at 2.67 h (mean % of daily calories = 27%), 4.42 h (28%), 12.42 h (31%), and 14.42 h (13%) after waking. Following another 8-h sleep episode, participants started a second CR (~32–55 h), followed by a 10-h recovery sleep episode before discharge.

### Blood sampling

Starting on Day 2, an indwelling, intravenous catheter was inserted in each participant’s forearm vein and plasma samples were collected every 20–60 min. Plasma samples collected during CR1 and CR2 were previously assayed for melatonin^25^, and re-assayed by a CLIA certified laboratory blind to the conditions of the experiment for either a lipid panel (total cholesterol, HDL-C, LDL-C, triglycerides, CHOL/HDL-C ratio, non HDL-C; Test code: 7600, Quest Diagnostics, LLC Marlborough, MA) or hepatic protein panel (total protein, albumin, globulin, and albumin/globulin ratio) (Test code: 90843, Quest Diagnostics, LLC Marlborough, MA), every 1–2 h such that every other sample was assayed for the same panel. The assays measured directly were total protein, albumin, total cholesterol, triglycerides, and HDL-C. Globulin and LDL-C were calculated by Quest Diagnostics (LLC Marlborough, MA) using clinically accepted standards.

### Data analysis

All statistical analyses were conducted in SAS 9.4 (SAS Inc., Cary, NC, USA) and graphical representations were produced using Prism 8.4 (GraphPad Software, La Jolla CA, USA). As reported in ref. ^25^, three participants were excluded for protocol compliance/errors and two additional participants were excluded because there was insufficient data to accurately assess the phase of melatonin. For the present study, we only analysed data from the 16 participants (8F, mean age ± SD 24.00 ± 3.16 years) who were included in the construction of the PRC for shifts in melatonin^25^.

There were a total of 1795 plasma samples assayed for lipids and hepatic proteins (7 of which could not be assayed). The 1795 samples were the same plasma samples previously analysed for melatonin (1 of which could not be assayed). In total, 27 samples (1.50%) were removed prior to analysis of phase shifts and PRC construction because of abnormal concentrations (e.g., out of the range of detection or values being outside of normative).

### Circadian rhythm analysis

Rhythms were assessed using a cosinor regression of the form^41^:

1 $$y=\mu +A\left({{\cos }}\left(\frac{2{{{{{\rm{\pi }}}}}}\left(x-\phi \right)}{24}\right)\right)$$

where *y* is the assay result (i.e., concentration), *x* is the clock time of the sample, *μ* is the mesor, and *ϕ* is the acrophase. The cutoff used for statistical significance was *p* < 0.1 (two-tailed).

### Phase response curve

Phase shifts were calculated as the difference between initial phase (CR1 acrophase) and final phase (CR2 acrophase) of the rhythms. Per convention, phase delays were plotted as negative values and phase advances as positive values on the ordinate.

The PRC was fitted with a single-harmonic function of the form:

2 $$y=\mu +A\left(\frac{\pi {{\sin }}(x-\phi )}{12}\right)$$

where *x* is circadian phase and *y* is phase shift. Parameters *μ*, *A*, *ϕ* represent the mean phase shift, amplitude, and phase, respectively, and pseudo-*r*^2^ and 95% confidence intervals were computed from the resulting fitted function. Statistical significance was determined using the overall fit of the nonlinear regression (PROC NLIN). Additional methods are provided in the Supplementary Materials.

### Reporting summary

Further information on research design is available in the Nature Research Reporting Summary linked to this article.

## Supplementary information

Supplementary Info

Reporting Summary

## Data Availability

The minimum dataset necessary to interpret, verify and extend the research in this article is available within the manuscript and its supplementary information. Source data are provided with this paper. De-identified individual data for all outcomes are provided in the Harvard Dataverse repository (10.7910/DVN/YADCK8)^42^.

## Source data

Source Data
