# Publisher Correction: Circadian lipid and hepatic protein rhythms shift with a phase response curve different than melatonin

**DOI:** 10.1038/s41467-022-29917-x

**Published:** 2022-04-20

**Authors:** Brianne A. Kent, Shadab A. Rahman, Melissa A. St. Hilaire, Leilah K. Grant, Melanie Rüger, Charles A. Czeisler, Steven W. Lockley

**Affiliations:** 1 Division of Sleep and Circadian Disorders, Departments of Medicine and Neurology, Brigham and Women’s Hospital, Boston, MA USA; 2 Division of Sleep Medicine, Harvard Medical School, Boston, MA USA; 3 Department of Psychology, Simon Fraser University, Burnaby, BC Canada

**Keywords:** Circadian mechanisms, Circadian regulation

Correction to: *Nature Communications* 10.1038/s41467-022-28308-6, published online 03 February 2022.

The original version of this article contained an error in the Abstract, where the text ‘0000’ was inadvertently introduced by the publisher. The incorrect sentence read ‘The timing of plasma rhythms was assessed by constant routine before and after exposure to a combined 6.5-hour blue light exposure and standard meal schedule, which was systematically varied by ~20° between in0000dividuals.’ The correct sentence now reads ‘The timing of plasma rhythms was assessed by constant routine before and after exposure to a combined 6.5-hour blue light exposure and standard meal schedule, which was systematically varied by ~20° between individuals.' This has now been corrected in the HTML and PDF versions of the article.

