# Supplementary material for: Circadian lipid and hepatic protein rhythms shift with a phase response curve different than melatonin
Source: Nat Commun. 2022 Feb 3;13:681. doi: 10.1038/s41467-022-28308-6 (PMC8814172; doi:10.1038/s41467-022-28308-6)
Supplement: Supplementary file 1 — Supplementary Info [file 41467_2022_28308_MOESM1_ESM.pdf]

Supplementary Information File

Supplementary Figures

Supplementary Figure 1

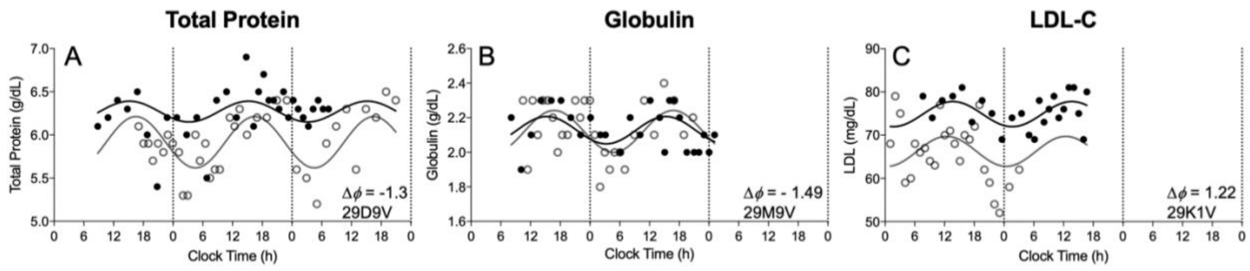

Supplementary Figure 1. Additional examples of rhythms during CR1 and CR2. Data collected during CR1 (black dots) and CR2 (grey open circles) for (A) total protein, (B) globulin, and (C) LDL-C. The x-axis is the time each blood sample was taken and the y-axis is the clinical assay concentration. The solid black line is the fitted cosinor regression for CR1 and the grey solid line is the fitted cosinor regression for CR2. The phase shift (Δϕ) and participant ID for each example is noted in the bottom right corner of the plots.

Supplementary Figure 2

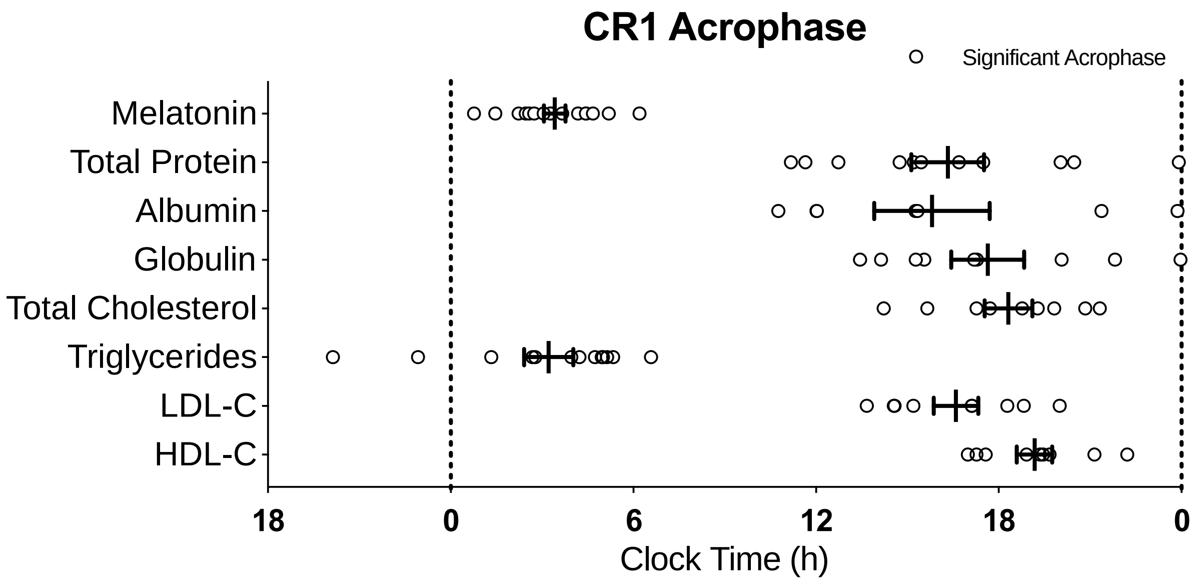

Supplementary Figure 2. Timing of CR1 acrophases. Each open circle represents data from one participant and indicates the timing of the acrophase of the statistically significant circadian rhythm for the marker listed along the y-axis. Black lines represent mean ± SEM.

Supplementary Figure 3

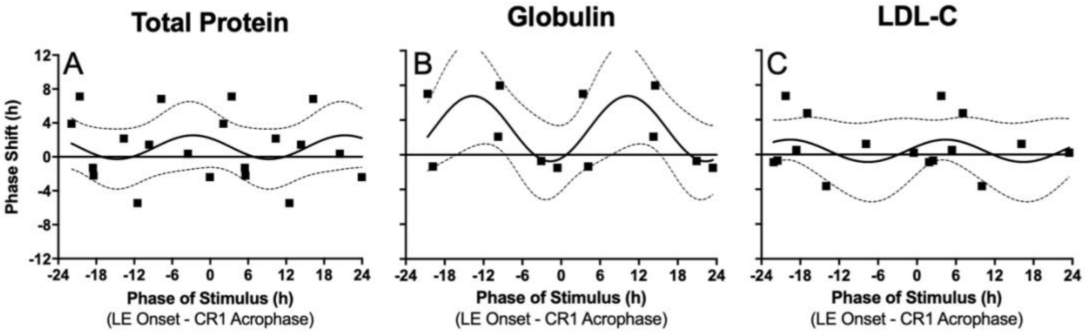

Supplementary Figure 3. Clinical assays that did not exhibit Phase Response Curves (PRCs). Raw phase shifts in (A) total protein, (B) globulin, and (C) LDL-C plotted as a function of circadian phase of the stimulus, defined as the onset of LE - CR1 assay-specific acrophase. Data are double-plotted. The solid horizontal black line indicates no phase shift. Black squares represent each individual who had statistically significant cosinor regressions in the assay during both CR1 and CR2. The solid black line is the fitted single-harmonic function. The grey dotted lines are the 95% confidence intervals generated from the fit single-harmonic function.

Supplementary Figure 4

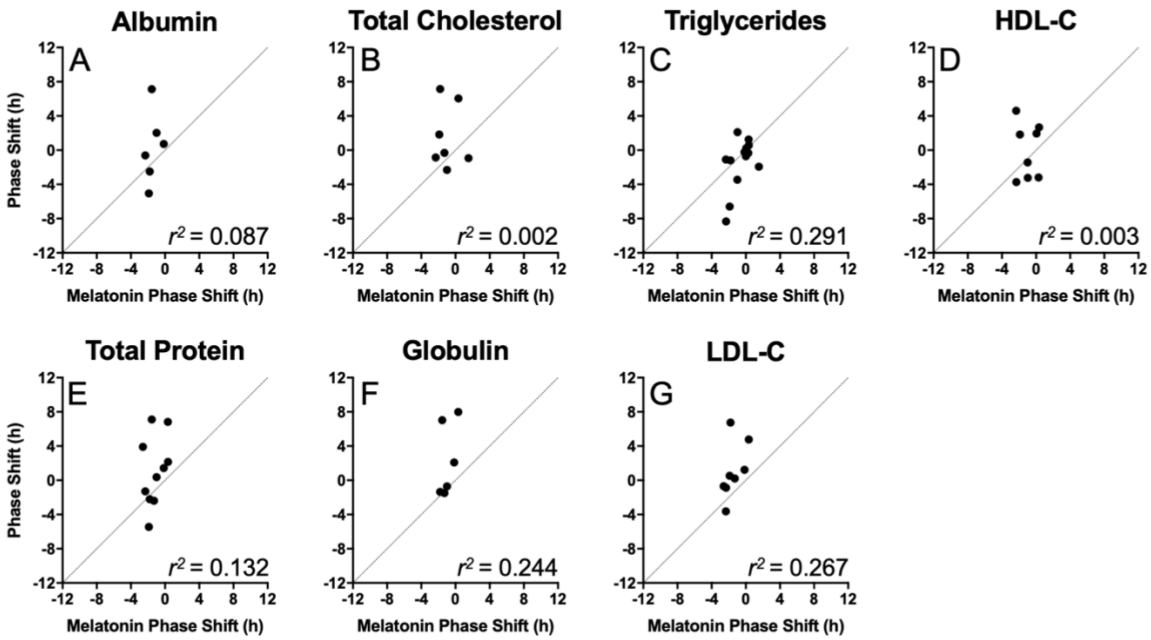

Supplementary Figure 4. Scatterplots illustrating the relationship between the phase shifts in melatonin and phase shifts in metabolic rhythms. Phase shifts of melatonin were plotted on the x-axis and phase shifts of (A) albumin, (B) total cholesterol, (C) triglycerides, and (D) HDL-C were plotted on the y-axis. Each black dot represents data from one participant. The diagonal dotted line represents r = 1.

Supplementary Figure 5

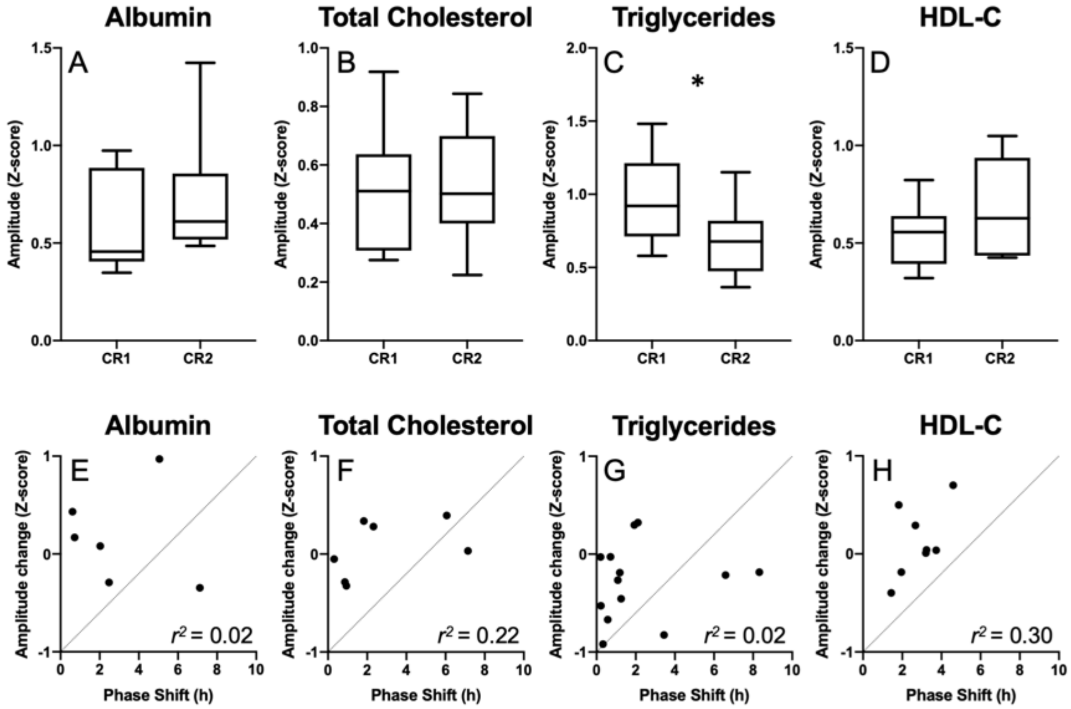

Supplementary Figure 5. Amplitude Analysis. Box plots showing the median and range of the amplitude of CR1 and CR2 z-scored data for (A) albumin, (B) total cholesterol, (C) triglycerides, and (D) HDL-C. Number of pairs (statistically significant rhythms during CR1 and CR2) included in the analyses (A) albumin = 6, (B) total cholesterol = 7, (C) triglycerides = 13 (p=0.02), and (D) HDL-C = 8. The bounds of the whiskers show the minimum and maximum values, the box represents the 25th to 75th percentiles and the median is represented by the horizontal line across the box. Scatterplots of the change in amplitude of the z-scored data plotted relative to phase shift for (E) albumin, (F) total cholesterol, (G) triglycerides, and (H) HDL-C. Each black dot represents data from one participant. The diagonal dotted line represents r = 1. * paired t-test p < 0.05

Supplementary Figure 6

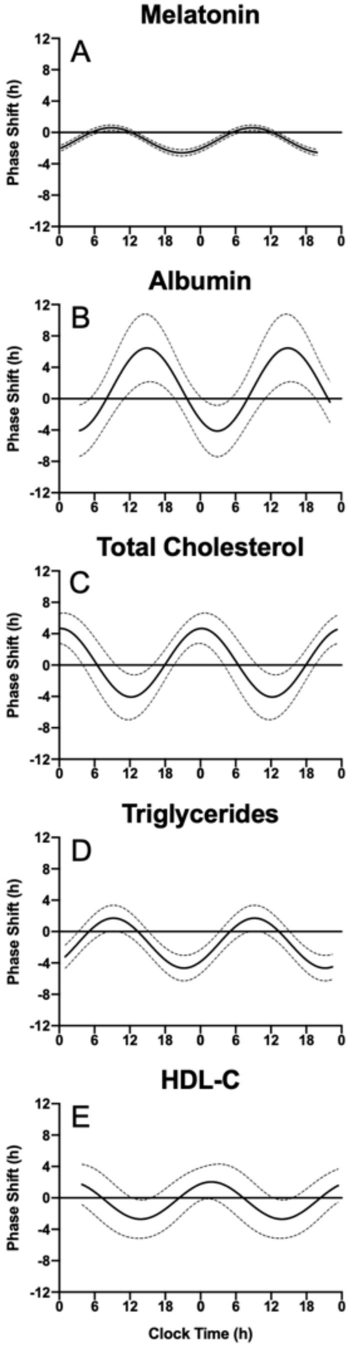

Supplementary Figure 6. PRCs adjusted to the group average CR1 acrophase. The fitted single-harmonic function of the PRC for (A) melatonin, (B) albumin, (C) total cholesterol, (D) triglycerides, and (E) HDL-C. PRCs are double-plotted with circadian phase of the stimulus on the x-axis, defined as the onset of LE relative to the CR1 assay-specific acrophase.

Supplementary Tables

Supplementary Table 1. Summary of statistically significant rhythms during CR1 for each participant

Participant	1	2	3	4	5	6	7	8	9	10	11	12	13	14	15	16
Melatonin	■	■	■	■	■	■	■	■	■	■	■	■	■	■	■	■
Total Protein	■	■	■	■	■	■	■	■	■	■			■			
Albumin	■	■	■	■		■	■						■			
Globulin		■		■	■	■	■	■	■	■			■			
Total Cholesterol	■	■	■	■	■			■		■	■				■	
Triglycerides	■	■	■	■	■	■	■		■		■	■		■	■	■
LDL-C	■		■	■	■	■		■		■	■	■				
HDL-C	■	■	■		■		■		■		■	■		■		

Shading indicates a statistically significant circadian rhythm during CR1

Supplementary Table 2. PRC fits with circadian phase of stimulus defined by DLMO or CR1 acrophase

Class	Assay	LE onset relative to DLMO			LE onset relative to CR1 acrophase		
		r^2	p	Amplitude	r^2	p	Amplitude
	Melatonin	0.76	0.0001	1.51	0.80	0.0001	1.60
Hepatic Proteins	Total Protein	0.14	0.27	2.18	0.05	0.66	1.40
	Albumin	0.07	0.70	1.66	0.60	0.02	5.29
	Globulin	0.45	0.07	3.66	0.32	0.17	3.76
Lipids	Total Cholesterol	0.25	0.20	2.46	0.70	0.001	4.37
	Triglycerides	0.50	0.0004	3.04	0.50	0.0004	3.18
	LDL-C	0.12	0.43	2.05	0.07	0.63	1.29
	HDL-C	0.61	0.002	3.12	0.39	0.04	2.37

Statistical significance was determined using the overall fit of the nonlinear (single harmonic) regression (PROC NLIN) ($p<0.05$, two-tailed).

Supplementary Methods

Participants

Participants were screened for medical and psychological health via examination, questionnaires, interview, and comprehensive urine and blood tests (1). Routine ophthalmology examinations were performed before and after the study. The absence of color-blindness was confirmed before the study using the Ishihara test (2). Participants kept a self-selected sleep-wake schedule of 8 h of sleep and 16 h of wake for at least three weeks prior to admission, that was verified by calls to a time- and date-stamped voicemail in addition to actigraphy for at least one week prior to admission (Actiwatch-L, Philips-Respironics, The Netherlands). Participants abstained from caffeine, nicotine, alcohol, and other restricted substances from the beginning of screening until completion, and compliance was evaluated by toxicology tests. Female participants were confirmed to be non-pregnant. Female participants not using oral contraceptives were tested in the luteal phase of their menstrual cycle (based on menstrual history), and female participants taking oral contraceptive were tested during times when they took a contraceptive with a stable hormone concentration.

Light Exposure

The monochromatic light source was generated by using a 1200-W xenon arc lamp and a grating monochromator. Power readings were taken regularly throughout the light exposure to ensure constant irradiance, using an IL-1400 radiometer with an SEL-033/F/W detector (International Light, Inc., Peabody, MA) by fixing the light meter to the

front of the dome at approximately eye level. The average corneal irradiance achieved during the light exposure was 11.75 ± 0.16 μW/cm2 (mean ± SD).

From 95 min prior to and throughout the light exposure, participants remained seated, and 15 min prior to the start of the light exposure, one drop of 0.5% cyclopentolate HCl (Cyclogyl, Alcon Laboratories, Fort Worth, Texas) was administered in each eye to dilate the pupils (3). Participants wore black-out goggles after the pupil dilator was administered until the start of the light exposure. Throughout the monochromatic light exposure, participants were asked to keep their gaze fixed for 90 min, while resting their head on a chin rest, before changing to a free gaze for 10 min. This was repeated throughout the exposure. Participants were monitored continually by a technician to ensure compliance. Light timing was randomized to one of 18 circadian phases separated by 80 min (≈20° intervals) according to habitual wake-time. Inter-individual differences in phase angle of entrainment contributed to a slightly varied interval of final light exposure phases.

Supplementary References

1. M. Ruger et al., Human phase response curve to a single 6.5 h pulse of short-wavelength light. J Physiol 591, 353-363 (2013).
2. S. Ishihara, The series of plates designed as a test for colour-deficiency. (Kanehara + Co., Ltd., Tokyo, Japan, 1996).
3. J. R. Gaddy, M. D. Rollag, G. C. Brainard, Pupil size regulation of threshold of light-induced melatonin suppression. J Clin Endocrinol Metab 77, 1398-1401 (1993).
