# Supplementary material for: Circadian lipid and hepatic protein rhythms shift with a phase response curve different than melatonin
Source: Nat Commun. 2022 Feb 3;13:681. doi: 10.1038/s41467-022-28308-6 (PMC8814172; doi:10.1038/s41467-022-28308-6)
Supplement: Supplementary file 2 — Reporting Summary [file 41467_2022_28308_MOESM2_ESM.pdf]

nature research

Corresponding author(s): Steven Lockley

Last updated by author(s): Aug 3, 2021

Reporting Summary

Nature Research wishes to improve the reproducibility of the work that we publish. This form provides structure for consistency and transparency in reporting. For further information on Nature Research policies, see our Editorial Policies and the Editorial Policy Checklist.

Statistics

For all statistical analyses, confirm that the following items are present in the figure legend, table legend, main text, or Methods section.

n/a	Confirmed	
☐	☒	The exact sample size (n) for each experimental group/condition, given as a discrete number and unit of measurement
☐	☒	A statement on whether measurements were taken from distinct samples or whether the same sample was measured repeatedly
☐	☒	The statistical test(s) used AND whether they are one- or two-sided<br>*Only common tests should be described solely by name; describe more complex techniques in the Methods section.*
☒	☐	A description of all covariates tested
☐	☒	A description of any assumptions or corrections, such as tests of normality and adjustment for multiple comparisons
☐	☒	A full description of the statistical parameters including central tendency (e.g. means) or other basic estimates (e.g. regression coefficient) AND variation (e.g. standard deviation) or associated estimates of uncertainty (e.g. confidence intervals)
☐	☒	For null hypothesis testing, the test statistic (e.g. F, t, r) with confidence intervals, effect sizes, degrees of freedom and P value noted<br>*Give P values as exact values whenever suitable.*
☒	☐	For Bayesian analysis, information on the choice of priors and Markov chain Monte Carlo settings
☒	☐	For hierarchical and complex designs, identification of the appropriate level for tests and full reporting of outcomes
☐	☒	Estimates of effect sizes (e.g. Cohen's d, Pearson's r), indicating how they were calculated

Our web collection on statistics for biologists contains articles on many of the points above.

Software and code

Policy information about availability of computer code

Data collection	No software was used
Data analysis	All statistical analyses were conducted in SAS 9.4 (SAS Inc., Cary, NC, USA) and graphical representations produced using Prism 8.4 (GraphPad Software, La Jolla CA, USA).

For manuscripts utilizing custom algorithms or software that are central to the research but not yet described in published literature, software must be made available to editors and reviewers. We strongly encourage code deposition in a community repository (e.g. GitHub). See the Nature Research guidelines for submitting code & software for further information.

Data

Policy information about availability of data

All manuscripts must include a data availability statement. This statement should provide the following information, where applicable:

- Accession codes, unique identifiers, or web links for publicly available datasets
- A list of figures that have associated raw data
- A description of any restrictions on data availability

Source data are provided with this paper. Requests for access to de-identified data can be made to the corresponding author.

Field-specific reporting

Life sciences study design

All studies must disclose on these points even when the disclosure is negative.

Sample size	21 participants (10F, mean age ± SD: 23.10 ± 3.43 years). Sample size was based on previous studies using the same protocol (Czeisler et al., Science, 1989; Khalsa et al., J Physiol, 2003; St Hilaire et al., J Physiol, 2012)
Data exclusions	3 participants were excluded for protocol compliance/errors and 2 additional participants were excluded because there was insufficient data to accurately assess the phase of melatonin
Replication	We removed Figure 4 from the revision, which included a partial replication. No replication efforts are reported here but we have found that these rhythms are consistent between multiple studies, which gives us additional confidence in these findings. To date, we have conducted three other studies with an additional 42 participants and observe similar results in rhythm timing. One study has been recently published: Grant, L. K., St Hilaire, M. A., Brainard, G. C., Czeisler, C. A., Lockley, S. W., & Rahman, S. A. (2021). Endogenous circadian regulation and phase resetting of clinical metabolic biomarkers. Journal of Pineal Research, e12752.
Randomization	The experimental methods were identical between individuals except that the timing of the 16-h combined light/meals exposure day was systematically varied to schedule the midpoint of the day 80 min (~20 degrees) apart between participants to collectively cover all circadian phases. Participants were randomly assigned to condition (the randomness is not noted in the manuscript but is noted in the initial manuscript Ruger et al., 2013).
Blinding	Complete blinding was not possible because the lab technicians (providing meals and conducting blood draws) needed to be aware of the unique schedule for each participant, but they were unaware of the hypothesized impact of the schedules. The participants were in a room free of obvious time cues, so they were blinded to their unique condition.

Reporting for specific materials, systems and methods

We require information from authors about some types of materials, experimental systems and methods used in many studies. Here, indicate whether each material, system or method listed is relevant to your study. If you are not sure if a list item applies to your research, read the appropriate section before selecting a response.

Materials & experimental systems

n/a	Involved in the study	
☒	☐	Antibodies
☒	☐	Eukaryotic cell lines
☒	☐	Palaeontology and archaeology
☒	☐	Animals and other organisms
☐	☒	Human research participants
☒	☐	Clinical data
☒	☐	Dual use research of concern

Methods

n/a	Involved in the study	
☒	☐	ChIP-seq
☒	☐	Flow cytometry
☒	☐	MRI-based neuroimaging

Human research participants

Policy information about studies involving human research participants

Population characteristics	Twenty-one healthy adults (10F, mean age ± SD: 23.10 ± 3.43 years). Participants were screened for medical and psychological health via examination, questionnaires, interview, and comprehensive urine and blood tests.
Recruitment	Participants were recruited from the community by posting flyers on university campuses and other sites, and newspaper advertisements. Our recruitment procedures provide all applicants with an equal opportunity to participate in our studies regardless of sex, race, color, creed, or national origin. We don't expect self-selection to bias the results as the participants are highly screened for medical and psychological health via examination, questionnaires, interview, and comprehensive urine and blood tests.
Ethics oversight	The study was approved by the Partners Human Research Committee at Brigham and Women's Hospital, in compliance with the Declaration of Helsinki. All participants gave written informed consent prior to enrolling in the study and were paid for their participation.

Note that full information on the approval of the study protocol must also be provided in the manuscript.
